# Metabolic Dysregulation Contributes to the Progression of Alzheimer’s Disease

**DOI:** 10.3389/fnins.2020.530219

**Published:** 2020-11-05

**Authors:** Xu Yan, Yue Hu, Biyao Wang, Sijian Wang, Xinwen Zhang

**Affiliations:** ^1^The VIP Department, School and Hospital of Stomatology, China Medical University, Liaoning Provincial Key Laboratory of Oral Diseases, Shenyang, China; ^2^Center of Implant Dentistry, School and Hospital of Stomatology, China Medical University, Liaoning Provincial Key Laboratory of Oral Diseases, Shenyang, China

**Keywords:** Alzheimer’s disease, glucose metabolism dysregulation, glycolysis dysfunction, TCA cycle and OXPHOS deficits, pentose phosphate pathway impairment

## Abstract

Alzheimer’s disease (AD) is an incurable neurodegenerative disease. Numerous studies have demonstrated a critical role for dysregulated glucose metabolism in its pathogenesis. In this review, we summarize metabolic alterations in aging brain and AD-related metabolic deficits associated with glucose metabolism dysregulation, glycolysis dysfunction, tricarboxylic acid (TCA) cycle, oxidative phosphorylation (OXPHOS) deficits, and pentose phosphate pathway impairment. Additionally, we discuss recent treatment strategies targeting metabolic defects in AD, including their limitations, in an effort to encourage the development of novel therapeutic strategies.

## Introduction

Alzheimer’s disease (AD) is an age-related neurodegenerative disease characterized by a progressive loss of neuronal structure and function. It is the most common type of dementia worldwide. The condition involves a progressive deterioration in memory, cognition, and mobility (Alzheimer’s Association, 2012). Its pathophysiology is extremely complex and heterogeneous, entailing accumulation of senile plaques caused by abnormal amyloid β (Aβ) metabolism, and neurofibrillary tangles caused by tau hyperphosphorylation (i.e., the formation of p-tau). Furthermore, the cerebrovascular system is seriously damaged, including the disturbance of the blood-brain barrier (BBB) and cerebral amyloid angiopathy (Viswanathan and Greenberg, 2011). Increased levels of reactive oxygen species (ROS) induce the transcription of pro-inflammatory genes and the release of cytokines (e.g., interleukin-1β [IL-1β], IL-6, and tumor necrosis factor-alpha [TNF-α]) and chemokines that cause neuroinflammation. In addition, reactive microglia and astrocytes and other pathological events also contribute to the dysfunction and deprivation of synapses and, ultimately, neuronal death (Martins et al., 2018). Aging is a major risk factor for AD. Some similarities and differences occur in glucose metabolism-related proteins have been observed in the brain during normal aging and AD (Klosinski et al., 2015). The apolipoprotein E (ApoE) gene is responsible for synaptic repair and neuronal structure maintenance and is a major risk factor for the sporadic form of AD. Of the three major isoforms (i.e., ApoE2, ApoE3, and ApoE4), people with the ApoE4 allele are at higher risk of developing AD than the others (Munoz and Feldman, 2000).

The brain consumes the greatest amount of energy of all the organs in the body. Because neurons require large amounts of energy to maintain their normal activities, a metabolic decline in the aging brain contributes to cognitive impairment (Boveris and Navarro, 2008). There is an age-related decrease in glucose utilization in most human brains (Petit-Taboué et al., 1998). In addition, reduced O_2_ uptake has been observed in the aging rodent brain (Navarro and Boveris, 2008). The pathological metabolic alterations in aging (e.g., cerebral glucose hypometabolism) are early and consistent events in the progression of AD. Glucose, the main transportation form of carbohydrate in our blood, is also the crucial and primary energy substrate for the brain under physiological conditions (Bouzier-Sore et al., 2006). However, alternative substrates, such as glycogen, ketone bodies, and amino acids, are also critical. Energy hypometabolism, particularly a decline in glucose metabolism, is one of the earliest and most common anomalies observed in patients with AD (Small et al., 2006). Indeed, insulin and insulin growth factor-1 (IGF-1) signaling help to maintain and control metabolism and cognition in the central nervous system (CNS) (de la Monte and Wands, 2005), and insulin resistance is one of the main risk factors for AD (Diehl et al., 2017). The main intracellular energy metabolism pathways occurring in our brains are complicated and include anaerobic glycolysis and the pentose phosphate pathway (PPP) in the cytoplasm, as well as oxidative phosphorylation (OXPHOS) in mitochondria and the tricarboxylic acid (TCA) cycle (also known as the citric acid cycle and Krebs cycle) (Dienel, 2019).

Metabolic processes are regulated by a series of key enzymes. Indeed, a growing body of evidence suggests the presence of organic impairment of mitochondria (Macdonald et al., 2018) and damage to related metabolic enzymes (Bubber et al., 2005). In addition, oxygen and glucose metabolic rates are drastically changed in many neurodegenerative diseases, including AD due to marked alterations in the glycolytic pathway and TCA cycle (Hoyer, 1982; Arias et al., 2002; van Gijsel-Bonnello et al., 2017). To add insult to injury, metabolism dysregulation is related to inflammatory responses, particularly in microglia. Baik et al. (2019) reported that Aβ could directly activate microglia to produce inflammatory factors by shifting their metabolism from OXPHOS to aerobic glycolysis. Thus, understanding the relationship between dysfunctional metabolism and AD could provide new insights into the pathogenesis of AD to support the development of new therapies. In this review, we discuss normal metabolic processes, aging, AD-related dysregulation, and relevant treatment strategies.

## Glucose Metabolism Dysregulation

### Normal Glucose Metabolism

As the major energy source for the brain, glucose is metabolized to ATP, an unstable high-energy compound. Glucose metabolism in the brain involves several stages. First, a signal is received by the brain to trigger glucose uptake (e.g., insulin signaling). Next, the physiological process of glucose uptake occurs. This process is dependent on glucose transporters (GLUTs) spread throughout the brain that allow glucose to cross the BBB and reach the neurocytes (e.g., astrocytes) (Molofsky et al., 2012). There are several different GLUT subtypes in the brain. GLUT1 (55 kDa) transfers glucose from the blood into the extracellular space of the brain through the BBB endothelium, while GLUT1 (45 kDa) and GLUT3 take up glucose into astrocytes and neurons, respectively (Cunnane et al., 2011). The insulin-sensitive GLUT4 is found in discrete subsets of neurons (Choeiri et al., 2002). GLUT8 is located in the intracellular compartment of hippocampal and cerebellar neurons regulated by hormone, while its exact location and function are still undefined (Gomez et al., 2010). After uptake, glucose is metabolized through the glycolysis pathway to pyruvate, generating ATP. Finally, pyruvate is converted to acetyl coenzyme A (acetyl-CoA) via the TCA cycle, which eventually forms an electro-gradient to drive the rotation of the V-ATPase machinery, and energy is transferred from the electro-potential to the bound ATP (Adeva-Andany et al., 2016). The hydrogen generated from this oxidation is transformed into water and ATP through complexes I, II, III, and IV of the electron transport chain (ETC). This OXPHOS reaction occurs on the inner mitochondrial membrane (IMM) (Hauptmann et al., 2009). Concurrently, the carbon dioxide produced by decarboxylation is transported through the blood to the respiratory system and expelled.

Blood glucose accesses the brain via the GLUTs with the help of insulin (Arnold et al., 2018). Decreased expression of insulin-sensitive GLUTs is strongly associated with a decline in glucose uptake (Taguchi et al., 2007). Insulin and the insulin receptor (IR) are vital factors in regulating glucose utilization and energy homeostasis between the CNS and peripheral circulatory system. Insulin signaling is reciprocally linked to the mammalian target of rapamycin (mTOR) pathway via the phosphoinositide-3-kinase (PI3K)/Akt axis (O’Neill, 2013). As an intracellular energy sensor, mTOR is activated by growth factors, including amino acids and high cellular energy status. Thus, the mTOR signaling pathway plays a major role in regulating cell growth and lipid and glucose metabolism (Di Domenico et al., 2018). Additionally, both inhibited insulin signaling and altered protein homeostasis in early AD can lead to aberrant mTOR activation (Dennis et al., 2001). The coupling of PI3K/Akt to IGF-1 and IR can be eliminated through serine phosphorylation of insulin receptor substrate-1 (IRS-1), mediated by mTOR, and IRS-1 inactivation and degradation, which is a prominent trigger of brain insulin resistance (BIR) (Tanti and Jager, 2009). Insulin also downregulates mTOR due to continuous activation of IRS-1 by mTOR (Yoon, 2017). The insulin signaling cascade is also regulated by the unique Ser/Thr/Tyr kinase biliverdin reductase-A (BVR-A). Oxidative stress-induced impairment of BVR-A kinase activity is an early event. Moreover, glucose starvation (hypoglycemia) reduces the intracellular ATP/AMP ratio, activating AMP-activated protein kinase (AMPK). As the main sensor of intracellular fuel status activated by energy stress (Gowans and Hardie, 2014), AMPK participates in the induction of several genes responsible for the growth, maintenance, and repair of neuronal cells and synapses. In addition, AMPK can regulate the plasticity of the hippocampal synapse, the cornerstone of learning and memory (Akter et al., 2011). To sum up, GLUTs are regulated by multiple pathways, and a balance among these pathways is crucial to maintaining the stability of energy metabolism.

### Altered Glucose Metabolism in Aging and AD

The brain is mainly composed of terminally differentiated neurons. During aging, neurons with relatively low regenerative ability are unable to adapt to alterations in the basal metabolic rate, and the energy-driven state is decreased or degenerated, which may contribute to various neurodegenerative diseases (Swerdlow, 2007; Isaev et al., 2019). ^18^F-fluorodeoxyglucose (FDG) and Pittsburgh Compound B (PIB) positron emission tomography (PET) (PIB-PET and FDG-PET, respectively) are suitable for detecting brain glucose uptake. Micro-FDG-PET scans show that cerebral glucose uptake in normal-aging humans (Pardo et al., 2007) and aged rats (Jiang et al., 2013) is declined compared to the youth. Moreover, the role of reduced cerebral glucose uptake in age-related cognitive impairment has been verified in clinical studies (Gejl et al., 2017). The expression of neuronal GLUTs, such as GLUT3 and GLUT4, significantly decreases with aging, while GLUT1 (55 KDa) expression in vascular endothelial cells decreases only slightly (Jais et al., 2016). BIR, resulting in the partial inactivation of insulin signaling and the impairment of PI3K/Akt and several downstream pathways, has been linked to aging (Barzilai and Ferrucci, 2012). Ultimately, these changes contribute to a variety of characteristics of normal aging in the body.

Compared to normal aging, a strong reduction in glucose consumption can be observed in AD (Kato et al., 2016). Alterations in glucose metabolism can affect the maintenance of neurotransmission and neuronal function and impact the ability to learn and memorize. Specific damage to cerebral glucose metabolism has been detected in the posterior cingulum cortex and temporoparietal cortices using FDG-PET (Bohnen et al., 2012). In contrast, glucose metabolism is relatively constant in areas of primary sensorimotor and visual cortices, basal ganglia, and the cerebellum in AD patients (Womack et al., 2011). Decreased glucose utilization may occur even before the clinical symptoms of AD based on a study involving mild cognitive impairment (MCI) subjects (Drzezga et al., 2003). Glucose uptake in the precuneus, an area of early Aβ deposition, is significantly decreased in individuals with the disease 10 years before the appearance of symptoms (Bateman, 2012). Tau mutants regulate mitochondrial trafficking by altering the fragmentation of mitochondria in neuronal cells (Kausar et al., 2018). Two different sites in human islet amyloid polypeptide sequence are sensitive to BACE1-mediated APP cleavage (Rulifson et al., 2016). Aβ may be relevant to the interaction of IR and the GLUTs (Rulifson et al., 2016). The disturbance of neurogenic glucose metabolism caused by impaired insulin signaling results in AD characteristics that parallel the pathophysiology of non-nervous tissues in type 2 diabetes mellitus. The density of the neuronal GLUT3 transporter is associated with local cerebral glucose utilization (Duelli and Kuschinsky, 2001). A reduction in GLUT1 and GLUT3 in AD patient brains (Simpson et al., 1994; An et al., 2018) is correlated with a decline in brain glucose consumption and cognition impairment (Landau et al., 2010). The decline may result from the abnormal hyperphosphorylation of tau and decreased hypoxia-inducible factor-1 α (HIF-1α) levels, which contribute to the transcriptional activation of GLUT (Liu et al., 2008). GLUT4 is reduced in male 3xTG-AD mice (Sancheti et al., 2013); however, cerebral glucose uptake does not coincide with GLUT4 expression in female mice. In addition, astrocyte activation may contribute to the GLUT2 upregulation observed in postmortem brain tissue from AD patients (Liu et al., 2008).

The insulin and IGF-1 signaling (IIS) pathway has considerable effects on metabolism regulation and cognitive function (de la Monte and Wands, 2005). IIS binds to tyrosine kinase receptors and IGF-1 receptor (IGF-1R), which are widely distributed in the hippocampus and cerebral cortex in AD (Freude et al., 2009). The insulin signal is inhibited in the AD brain, which is closely connected to inefficiency in glucose metabolism (Neth and Craft, 2017). The impairment of insulin signaling also contributes to abnormalities in mitochondrial structure and function (Cheng et al., 2010). Moreover, significant gene expression alterations observed in the AD brain are related to the generation and transmission of insulin signals (Hokama et al., 2014). For example, the post-synaptic β-aminobutyric acid (GABA) - a receptor accumulates on the cell surface rapidly when the insulin pathway is activated; however, the genes encoding GABA receptors are markedly reduced in AD brain tissue (Luscher et al., 2011). Insulin competitively inhibits the insulin-degrading enzyme (IDE) to degrade Aβ and elevates extracellular Aβ levels by promoting its secretion (Gasparini et al., 2001). IDE is involved in insulin function/resistance and metabolism-related processes and plays an important role in the degradation of Aβ monomers (Farris et al., 2004). However, its expression is reduced in AD patients (Craft et al., 2003). Energy-transducing pathways always occur in the mitochondria, and the initial activation of IIS requires mitochondria to produce low-level H_2_O_2_, which reflects the energy state of mitochondria and is involved in the regulation of redox (Storozhevykh et al., 2007). The insulin receptor (IR) and insulin receptor substrate (IRS) renders them susceptible to oxidation (and activation) by H_2_O_2_ (Yin et al., 2014). Excess H_2_O_2_ can give rise to BIR (Swerdlow, 2011), which is a risk factor for diabetes mellitus and AD (Luchsinger et al., 2001; Bedse et al., 2015). Thus, the regulation of redox-sensitive signals in the mitochondria should also be considered (Yin et al., 2014). BIR compromises the intracellular translocation of low-density lipoprotein receptor-related protein 1 (LRP1, a receptor for ApoE) to the plasma membrane of hepatocytes, potentially hindering the hepatic elimination of circulating Aβ (Tamaki et al., 2007). In a 3xTg-AD mouse model, oxidative stress first causes consistent activation of IRS-1 and then activates negative feedback mechanisms (e.g., mTOR) to disable IRS-1 hyperactivity and cause BIR (Barone et al., 2016). Overall, BIR is regulated by aberrant insulin signaling and may contribute to disordered glucose metabolism, oxidative stress, BBB dysfunction, and energy supply insufficiency, which are pathological features of diabetes mellitus that can further affect Aβ generation and clearance (Leuner et al., 2012; Salameh et al., 2016). Thus, insulin resistance that induces glucose hypometabolism might be the main cause of energy deficiency in AD brains, which resemble, but is distinct from, the manifestation of diabetes. Thus, it is hypothesized that AD is a neuroendocrine disorder that may be identified as “Type 3 diabetes,” reflecting a new mechanism of neurodegeneration (Steen et al., 2005).

As mentioned above, insulin mediates the phosphorylation of downstream mTOR by activating PI3K/Akt signal pathways in our brains. As one of the cellular degradation pathways of misfolded and unfolded proteins in neurodegeneration (Garcia-Arencibia et al., 2010), autophagy is regulated by mTOR, which affects ATP levels and biosynthetic pathways (Ulland et al., 2017). Because the maturation of autolysosomes and their retrograde transport are impeded in AD, a massive accumulation of defective autophagosomes can lead to autophagy intermediates (autophagic and lysosomal vesicles) within large swellings along dystrophic and degenerating neurites (Nixon, 2007). Defective autophagy leads to the overproduction, aggregation, and diminished clearance of Aβ and p-tau, oxidative damage, and mitochondrial dysfunction, which, in turn, contribute to the impairment of the metabolic pathways controlled by insulin and mTOR (Perluigi et al., 2015). Moreover, mTOR signaling hyperactivity inhibits the induction of the autophagy clearance system (Majumder et al., 2011; Orr and Oddo, 2013) and increases the accumulation of Aβ in AD (Cai et al., 2012; Perluigi et al., 2014). Thus, defective autophagy causes various effects in AD.

Triggering receptor expressed on myeloid cells 2 (TREM2) is one of the immune receptors expressed in the plasma membrane of microglia in the brain that can recognize phospholipids, lipoproteins, and apoptotic cells (Wolfe et al., 2018). Studies have shown that TREM2 defects can lead to impaired mTOR activation and the enhancement of AMPK activation in microglia in AD patients and an AD mouse model. This effect can result in hyperactive autophagy and microglial energy impairment, which can be compensated by energy repletion (Ulland et al., 2017). Thus, defective autophagy caused by abnormal mTOR regulation plays an important role in the development of AD. Thus, TREM2 represents a risk factor for AD (Ulland et al., 2017); however, the mechanism is unknown.

Moreover, decreased metabolic levels are more likely to be detected in the brains of ApoE4 carriers (Mosconi et al., 2004). ApoE4 is a crucial risk factor for late-onset AD (Zhao et al., 2018). There are key structural differences between the three isoforms of ApoE. ApoE2 has two cysteine residues (Cys, residues 112 and 158), while ApoE3 has a positively charged arginine residue at position 158, and ApoE4 has positively charged arginine residues at both positions 112 and 158 (Muñoz et al., 2019). The differences in the protein structures of the three ApoE isoforms affect their interactions with other proteins and peptides. Accumulating evidence has shown that ApoE, particularly ApoE4, binds to residues 12–28 of Aβ, and this binding promotes Aβ accumulation and strengthens Aβ pathology, especially oxidative stress (Ma et al., 1996; Potter and Wisniewski, 2012; Huang and Mahley, 2014; Liao et al., 2017). Oxidative stress-related modification analysis of plasma and cerebrospinal fluid (CSF) proteins demonstrated that ApoE oxidation could affect the antioxidant activity of thiol, which allows the formation of lipoprotein particles caused by excessive oxidative damage (Di Domenico et al., 2016). Under high levels of oxidative stress, lipid peroxidation produces the highly reactive and neurotoxic molecule, 4-hydroxynonenal (HNE), which covalently binds to Cys residues. Covalently modified Cys residues significantly alter the structure and function of modified proteins. HNE binds to Cys residues in ApoE2 and ApoE3, protecting other proteins from HNE damage. However, ApoE4 lacks Cys residues. Therefore, it cannot scavenge HNE, permitting this neurotoxic molecule to modify neuronal proteins and induce cell death (Sultana et al., 2013; Di Domenico et al., 2017). ApoE2 appears to have neuroprotective effects in the AD patient brain (Keeney et al., 2015) and exhibits the most robust metabolic profile for glucose uptake and glycolysis (Wu et al., 2018). In contrast, ApoE4 causes the most detrimental effects on aging and AD brains. There is reduced cerebral glucose metabolism in cognitively normal individuals carrying ApoE4 (Reiman et al., 2004, 2005). The brains of ApoE4 mice have lower GLUT3 mRNA levels compared to ApoE3 mice, which contributes to inadequate energy supplies in the ApoE4 brain (Wu et al., 2018). Understanding the metabolic process and its effects on the early or progressive alterations in AD may provide new treatment strategies for this irreversible neurodegenerative disease.

### Treatment

Several strategies are available for the development of therapeutics to prevent or slow down the progression of AD. Given that BIR is the main risk factor for AD, Reger et al. (2006) showed that a single dose of intranasal insulin can significantly improve the memory of patients with AD or mild cognitive impairment. Based on PET findings, intranasal insulin increases ^18^F-fluorodeoxyglucose uptake in the precuneus, frontal, cuneus, and parietotemporal regions of the brain (Craft et al., 2012). A Phase 2/3 multi-site clinical trial with intranasal insulin conducted between 2014 and 2018 showed no significant adverse reactions, and treatment improved cognitive function (Craft et al., 2020). Therefore, the specific regimen for intranasal insulin injection and its feasibility require further study. Moreover, one of the common drugs used in diabetes (peroxisome proliferator-activated receptors [PPAR]-γ agonists) increases metabolic efficiency by enhancing insulin sensitivity to reduce Aβ levels (Nicolakakis et al., 2008). Because the mTOR signaling pathway participates in multiple processes that regulate neuronal functions, rapamycin can be used to improve learning and memory and reduce Aβ and tau pathology (Caccamo et al., 2010). In addition, oral administration of rapamycin in a 3xTg-AD mouse model relieves memory symptoms (Spilman et al., 2010). Moreover, rapamycin analogs (Rapalogs), which have been approved by the FDA when used concurrently with metformin, are recommended to pharmacologically address the impaired glucose metabolism (Kezic et al., 2018).

Evidence suggests that short- and long-acting intranasal insulin therapy can improve memory in AD patients in an ApoE4-dependent manner (Reger et al., 2008; Claxton et al., 2015). In response to reduced glucose metabolism, mammalian cells promote the synthesis and utilization of ketone bodies, circulating energy sources for tissues in times of fasting or prolonged exercise (Akram, 2013). Targeted replacement of ApoE in mice with the human ApoE genes demonstrated that brains from ApoE2 and ApoE4 mice have stronger absorption and metabolism of ketone bodies than brains of ApoE3 mice (Wu et al., 2018). Moreover, ApoE^–^/^–^ mice (an AD model) exhibit circadian rhythm disturbances and increased tau deposition. These phenomena are related to energy shortage and degeneration of the suprachiasmatic nucleus, which can be alleviated by supplementing with ketone bodies in the absence of glucose (Zhou et al., 2016). Therefore, ApoE and ketone bodies may represent new therapeutic targets for improving brain energy metabolism in patients with AD. The specific mechanisms associated with ApoE and ketone bodies and their related treatment pathways need further investigation.

Earlier, we defined the important proteins needed for glucose to flow from the blood into the brain and subsequently regulate metabolic changes under normal conditions. We then summarized a series of changes in these proteins and other risk factors (e.g., ApoE4) in aging and AD. The insulin signaling pathway is closely linked to metabolic change, and the manifestation of decreased brain energy metabolism caused by insulin resistance is similar to diabetes. Thus, the “Type 3 diabetes” hypothesis is well supported. Furthermore, PET is a method used clinically to detect glucose intake and a reference for AD diagnosis. Given these metabolic changes, we described several potential clinical treatments; however, an effective treatment still requires further study.

## Glycolysis Dysfunction

### Normal Mechanism and Biological Function

Glycolysis, as the first step of glycometabolism and one of the main energy sources, plays an essential role in CNS metabolism. GLUT1 and GLUT3, mainly expressed on astrocytes and neurons, mediate the entry of glucose into these cells. Glucose can then be converted into glucose-6-phosphate (G6P) and then into fructose-6-phosphate (F6P) by hexokinase (Goncalves et al., 2018). F6P, which continues to participate in the subsequent steps of glycolysis, is converted into pyruvate. On the other hand, G6P not only participates in both glycogen synthesis and the PPP, but also non-competitively inhibits hexokinase (DiNuzzo et al., 2015). Phosphofructokinase (PFK), which takes part in the second step of glycolysis, catalyzes F6P into fructose 1,6-bisphosphate (F1,6BP). Fructose 2,6-bisphosphate (F2,6BP), which upregulates PFK-1, is generated via a reaction catalyzed by fructose 2,6-bisphosphatase isoform 3 (PFKFB) (Herrero-Mendez et al., 2009). Astrocytes are reported to have higher glycolysis activity due to higher PFK1 activity. In contrast, neurons exhibit lower glycolytic capacity due to lower levels of PFK1 and 6-phosphofructo-2-kinase and PFKFB (Bolanos et al., 2010). Thus, astrocytes have a significant effect on glycolysis in the CNS. In addition, the astrocytic levels of the abovementioned enzymes are elevated when rat hippocampal astrocytes are co-cultured with neurons, which suggests that neurons can affect astrocyte glycolysis (Mamczur et al., 2015).

Microglia maintain normal brain function by providing trophic support, respond to changes in CNS metabolism, and carry out classic immune cell functions that promote phagocyte clearance (Sarlus and Heneka, 2017; Ghosh et al., 2018). An *in vivo* study reported that the immune response of microglia is based on glycolysis metabolism (Baik et al., 2019). As first reported in 2002, the inflammasome, a multi-protein complex assembled by intracytoplasmic pattern recognition receptors, plays an important role in the natural immune system (Martinon et al., 2002). NLRP3 is the most studied component of the inflammasome. While the function of the NLRP3 inflammasome in astrocytes is controversial, it is known to be expressed and activated in microglia (Gustin et al., 2015; Freeman and Guo, 2017; Slowik et al., 2018). Evidence shows that pentabromophenol (PBP) and tetrabromobisphenol A (TBBPA) improves the metabolic rate of glycolysis in mouse microglia and promotes the activation of NLRP3 inflammasome (Bowen et al., 2020). Hexokinase is one of the key glycolytic enzymes. Wolf et al. (2016) reported that *N*-acetylglucosamine activates the inflammasome, particularly the NLRP3 inflammasome, through hexokinase inhibition by promoting its disassociation from the outer mitochondrial membrane (OMM).

### Altered Glycolysis in Aging and AD

Studies in mouse models of different ages found that the levels of glycolysis products (e.g., G6P and F1,6BP) decreased in adult mice compared to young mice (Hoyer, 1985). However, recent studies demonstrated that glycolysis increased with a decline in the resting cerebral blood flow of the aged brain (Lourenço et al., 2017). It also increased in naturally aging astrocytes (Cao et al., 2019). Furthermore, glycolysis dysfunction can lead to age-related neurodegeneration (Hipkiss, 2019). Therefore, glycolysis has a complex relationship with growth and aging in the brain.

Considerable research has examined the associations between AD and the enzymes intimately linked to glycolysis (i.e., hexokinase, glyceraldehyde 3-phosphate dehydrogenase [GAPDH], and pyruvate kinase [PK]) (Vallee et al., 2018b; Wu et al., 2018; Butterfield and Halliwell, 2019). Cisternas et al. (2019) reported that Wnt signaling promotes glucose metabolism by increasing the expression of hexokinase and PFK, which causes neuroprotective effects. However, decreased hexokinase and PFK expression and dysregulated Wnt signaling are observed in AD. The accumulation of G6P can reduce hexokinase activity by competitively inhibiting ATP binding to the active site of the enzyme (Liu et al., 1999). In addition, an unbiased metabolomics approach demonstrated G6P accumulation in humans and mice with AD, which restrains glycolysis (Demarest et al., 2020). Hexokinase binds to the OMM via the voltage-dependent anion channel (VDAC), which controls the mitochondrial permeability transition pore (MPTP) (Harris et al., 2014). When hexokinase associates with VDAC, the MPTP tends to be closed. In the postmortem brain tissue of AD mice and patients, hexokinase levels were decreased while VDAC1 levels were elevated (Cuadrado-Tejedor et al., 2011). Furthermore, the interaction of VDAC1 with hexokinase I can generate outer membrane potential in brain mitochondria. Outer membrane potential metabolic-dependent homeostasis can prevent the mitochondrial permeability transition, which leads to Ca^2+^ activation and neuronal cell death. In addition, this may be involved in resistance to neuronal death and neurodegenerative disorders such as AD (Lemeshko, 2018). Glycogen synthase kinase 3 (GSK3), a serine/threonine protein kinase, disassociates VDAC1 from hexokinase (Reddy, 2013). The activation of GSK3 promotes apoptosis in neuroblastoma cells by reducing the level of cyclin D1 (Kunnimalaiyaan et al., 2018), which inhibits the extrinsic apoptotic signaling pathway mediated by death receptor (Beurel and Jope, 2006). Tau phosphorylation, one of the characteristics of AD, is regulated by GSK3 (Hernandez et al., 2013). Notably, GSK3 inhibition attenuates the symptoms of mild cognitive impairment (Nunes et al., 2013) and restrains oxidative stress in AD (Vallee et al., 2017). Activation of the NLRP3 inflammasome resulting from mitochondrial DNA synthesis in macrophages results in damage to macrophage mitochondria (Zhong et al., 2018). The NLRP3 inflammasome-driven proinflammatory cascade in microglia is augmented by impaired mitochondrial function (Sarkar et al., 2017). Furthermore, NLRP3^–/–^ or caspase-1^–/–^ mice with AD-related mutations largely avoid spatial memory loss and other sequelae associated with AD, suggesting a key role for the NLRP3 inflammasome in the pathogenesis of AD (Heneka et al., 2013). Overall, the hexokinase dysfunction observed in AD brain samples is mediated by the accumulation of G6P that dissociates hexokinase from mitochondria. This process activates the NLRP3 inflammasome, indicating that G6P accumulation may contribute to neuroinflammation in AD. Therefore, the effect of hexokinase activity on glycolysis in the CNS and mitochondrial function is a starting point to study the metabolic mechanism of AD.

GAPDH participates in the sixth step of glycolysis, catalyzing glyceraldehyde 3-phosphate to 1,3-bisphosphoglycerate and increases the NADH:NAD^+^ ratio (Diaz-Garcia et al., 2017). 2-Deoxyglucose (2DG), which inhibits glycolytic processes (Kim et al., 2017), prevents neurodegeneration by eliminating microglia (microglia can damage neurons in an inflammatory situation). In addition, it reduces the impact of Aβ on neuron cells (Vilalta and Brown, 2014). Moreover, an increased NADH concentration reverses the abovementioned effects of 2DG (Shen et al., 2017). Hou et al. (Hou et al., 2018) found that a decreased NADH:NAD^+^ ratio is a possible way to reduce AD-associated pathology. Recent research also reported many other functions of GAPDH beyond glycolysis, including DNA repair (Kosova et al., 2017), control of the activity of kinase and phosphotransferase (Tisdale, 2002), and an integral association with membrane ion pumps participating in Ca^2+^ release (Patterson et al., 2005). Hyperphosphorylated tau protein leads to microtubule depolymerization, resulting in neuron dysfunction (Higuchi et al., 2005; Iqbal et al., 2005). Chen et al. (2000) showed that tau bound to denatured GAPDH but not the native molecule and that the aggregation of the non-native GAPDH in solution was ameliorated by tau. Nakajima et al. (2017) further reported that GAPDH aggregation induced by nitric oxide led to MPTP opening and cell death. An increase in GAPDH disulfide bonding in AD patients and transgenic AD mice compared with controls suggests a potential relationship between GAPDH disulfide bonding and protein aggregation (Cumming and Schubert, 2005). In addition, GAPDH promotes Aβ amyloidogenesis *in vitro* (Itakura et al., 2015). A recent study found that the concentration of *S*-glutathionylated-GAPDH in the blood of patients with AD was significantly higher than in the control group, indicating that this indicator is related to the degree of neuronal apoptosis during the progression of AD (Tsai et al., 2020). Thus, GAPDH and its substrates are associated with neuronal cell death in AD. Moreover, Aβ causes microglial inflammation and induces a shift in the metabolic pathway from OXPHOS to glycolysis in the 5XFAD mouse model. During this process, Aβ induces Akt phosphorylation to activate the mTOR-HIF-1α pathway. HIF-1α then increases GAPDH expression. Inhibition of this pathway decreases the levels of pro-inflammatory cytokines, including IL-1β and TNF-α. These data demonstrate the relationship between glycolysis and inflammation in microglia induced by Aβ (Baik et al., 2019).

PK, a rate-limiting enzyme in glycolysis, has four isomers: M1, M2, L, and R. PKM2, which controls the levels of glycolytic intermediates as well as ATP, is linked to neurodegenerative diseases (Vallee et al., 2018a). As described by the Warburg effect, upregulation of the Wnt/β-catenin pathway can promote glycolysis, which is connected to PKM2. In AD, the Wnt/β-catenin pathway is downregulated (partially via inactivation of PKM2) and this results in oxidative stress and cell death (Vallee et al., 2018b). An increase in PK2 expression has been reported in AD transgenic mouse models (Martire et al., 2016). PKM2 promotes cell proliferation by binding to receptors of activated growth factors and then induces dimer formation through phosphorylation (Christofk et al., 2008). In addition, a study that induced yeast pyruvate kinase Cdc19 to form amyloid-like aggregates *in vitro* found similar characteristics between Cdc19 and PKM2, its mammalian counterpart (Cereghetti et al., 2018). Thus, the association between PK and AD might be worthy of investigation.

### Treatment

The latest AD patient symptom management guidelines consider pimavanserin and citalopram to be the two most promising medications for AD (Kales et al., 2019). Pimavanserin selectively and inversely excites 5-hydroxytryptamine 2A (5-HT2A) receptor (Hunter et al., 2015). Citalopram prevents the reuptake of 5-hydroxytryptamine (5-HT) by inhibiting 5-HT transporter, blocking reuptake on the presynaptic membrane (El-Armouche et al., 2003). Thus, both pimavanserin and citalopram increase the concentration of 5-HT in the synaptic cleft. Tiritilli (2000) found that oxygen deficit and glucose deprivation inhibit the contractive response of umbilical arteries to 5-HT, which implies that aerobic glycolysis can increase sensitivity to 5-HT. Research into human pancreatic ductal adenocarcinoma also indicates that the levels of enzymes participating in glycolysis increase after 5-HT stimulation (Jiang et al., 2017). Furthermore, conscious mice injected with 5-HT exhibit an increased brain glucose concentration (Leonard, 1975). In addition, 5-HT facilitates glycolysis through PKM2 upregulation in breast cancer cells (Sola-Penna et al., 2019). Therefore, an increased concentration of 5-HT stimulates glycolysis, although the specific glycolysis-related mechanism in the CNS remains unclear.

Besides the international guidelines for AD treatment, other recent guidelines, such as Chinese guidelines for the treatment of AD, propose cholinesterase inhibitors and excitatory amino acid receptor antagonist as well as certain traditional Chinese medicines for the treatment of AD (Group Chinese Dementia and Cognitive Impairment Writing Group, Association Special Committee on Cognitive Disorders of the Chinese Medical Doctors Association, 2018). The relationship between cholinesterase and glycolysis in CNS has been studied in the last century (Peiss et al., 1949). A study in 2013 indicated that carbachol (a cholinergic agonist) could increase fluxes in both glycolysis and OXPHOS in SH-SY5Y neuroblastoma cells (Lu et al., 2013). Glucose metabolism dysregulation can influence acetyl coenzyme A and indirectly retard the synthesis of acetylcholine (Slotkin et al., 1990). In addition, the activity of choline acetyltransferase (formed from choline and acetyl coenzyme A) is below normal in AD patients’ brains (Bowen et al., 1976). In AD treatment, cholinesterase inhibitors, such as donepezil, may help patients to control the symptoms of the disease (Matsunaga et al., 2018; Ruthirakuhan et al., 2018).

Excitatory neurotransmission, particularly that of glutamate, as well as the receptors involved, play essential roles in synaptic plasticity (Riedel et al., 2003) via *N*-methyl-D-aspartic acid (NMDA) receptors (NMDARs) (Collingridge et al., 2009). NMDAR stimulation facilitates glucose uptake and glycolysis in oligodendroglial cells (Saab et al., 2016). However, intense signal stimulation by glutamate would produce excitotoxicity, leading to CNS damage (Rothman and Olney, 1986) via excessive Ca^2+^ entry (Choi, 1987). A study of mouse astrocytes provided evidence that glutamate promotes glycolysis and damages mitochondrial aerobic capacity (Yan et al., 2017). Aβ has two forms, a soluble oligomeric form and an insoluble aggregate form. The former is primarily responsible for neurodegeneration and diminished synaptic function in AD (Barghorn et al., 2005), and it can interact with NMDARs, leading to mitochondrial Ca^2+^ overload and cell apoptosis (Alberdi et al., 2010). Memantine, as one of the uncompetitive NMDAR antagonists, has a strong voltage dependency (Alam et al., 2017). A study in a male Wistar rat model showed that memantine and lithium could reverse the decreased in IL-4 induced by oligomeric Aβ_1__–__42_, whereas lithium alone had no effect (Budni et al., 2017). Meta-analyses also indicate that memantine, particularly in conjunction with cholinesterase inhibitors, inhibits or slows the progress of AD symptoms (Tan et al., 2014; Kishi et al., 2017; Matsunaga et al., 2018). Therefore, NMDAR antagonism by memantine might prevent excessive glycolysis mediated by excitatory neurotransmission and the resultant excitotoxicity in AD.

In short, aging and AD are closely related to abnormal glycolysis in the nervous system. Dysfunction of the key enzymes involved in glycolysis can affect the generation of Aβ and tau, the activation of the NLRP3 inflammasome, and even mitochondrial function. A variety of drugs for the treatment of AD, backed by the existing guidelines, can regulate glycolysis in the nervous system. Moreover, controlling the activity of enzymes and products that regulate glycolysis (e.g., hexokinase, GAPDH, and G6P) could significantly improve the nervous system and may represent a new direction for AD treatment.

## TCA Cycle and Oxphos Deficits

### Normal Mechanism and Biological Function

The TCA cycle is the central hub for energy metabolism, macromolecule synthesis, and redox balance. In normal aerobic organisms, most cellular glucose is converted into pyruvate through glycolysis. Pyruvate is subsequently oxidized by PDH to acetyl-CoA, which is fed into the TCA cycle. One acetyl-CoA molecule can generate six molecules of NADH and two molecules of FADH through this pathway. The coenzyme NADH contains many electrons, which are transferred to the ETC on the IMM. Finally, ATP and H_2_O are generated. This process is called OXPHOS and also generates ROS as a byproduct to maintain cellular homeostasis. However, excessive ROS production may contribute to oxidative stress, with mitochondria as the first target (Rego and Oliveira, 2003). The body utilizes antioxidants (e.g., superoxide dismutase [SOD] and glutathione [GSH] to offset the adverse effects of ROS, Indo et al., 2015). However, the brain is an organ with high oxygen consumption and low antioxidant defenses. Thus, it is vulnerable to oxidative stress (Cobley et al., 2018). In addition, α-ketoglutarate (α-KG) is a TCA cycle intermediate that is transformed into glutamate and GABA by glutamate decarboxylase (GAD) and glutamate dehydrogenase. Reducing equivalents (i.e., NADH) generated from OXPHOS synthesize ATP in conjunction with glutamate and GABA, which helps maintain synaptic plasticity (Bak et al., 2006).

In addition to antioxidants, the TCA cycle products themselves affect the mitochondrial redox balance. Nicotinamide nucleotide transhydrogenase (NNT), NADP^+^-dependent isocitrate dehydrogenase (ICDH) 2, and malic enzyme can generate NADPH using the electrons from NADH (Metherell et al., 2016; Navarro et al., 2017; Meimaridou et al., 2018). All these proteins can affect redox activity in mitochondria (Yin et al., 2012). The redox state of mitochondria can regulate energy metabolism via the oxidization of several metabolic enzymes, including aconitase, α-ketoglutarate dehydrogenase (α-KGDH), malate dehydrogenase (Reed et al., 2008), and succinyl-CoA-3-oxoacid CoA transferase (SCOT) (Garcia et al., 2010), and complexes I (Cortes-Rojo et al., 2020), II (Jones et al., 2019), and V (Kramer et al., 2018). It can also regulate S-glutathione glycosylation, a modification that reflects the redox state of mitochondria by reversibly forming mixed disulfide bonds between protein cysteine sulfhydryl and GSH (Schafer and Buettner, 2001; Hill and Bhatnagar, 2012). Therefore, NADPH plays a crucial role in a series of processes mediating glucose metabolism as part of the redox-energy metabolism axis.

### Altered TCA Cycle and OXPHOS in Aging and AD

Proteomics analysis found that the dysregulation of TCA enzyme levels during aging includes the upregulation of malate dehydrogenase 1 (MDH1), fumarate dehydrogenase 1 (FH1), and subunits of NADH dehydrogenase, succinate dehydrogenase, and pyruvate dehydrogenase (PDH). Impaired TCA cycle metabolism is also associated with the downregulation of ICDH 1/2 and a subunit of succinyl-CoA ligase in the brains of aged mouse brains (Guo et al., 2020). Furthermore, a reduction in the metabolites from the TCA cycle has been measured in both aging and AD mouse models, while increases in the levels of the ceramides and sphingosine-1 phosphate (i.e., inflammatory metabolites) occurred in the aging group. The study also found that the levels of NADH and acetyl-CoA were positively correlated with age and the degree of AD, while glutamine and GABA concentrations were negatively correlated (Dong and Brewer, 2019). A decline in glutamine and GABA levels is associated with impaired neurotransmitter circulation (Dedeoglu et al., 2004; Tiwari and Patel, 2012). Moreover, acetyl-CoA and NADH^+^ have allosteric inhibitory effects on PDH, which subsequently affects the TCA cycle (Jha et al., 2016). Many studies have demonstrated that in the aging brain, as mutations in mitochondrial DNA (mtDNA) increase, the expression of respiratory chain complexes I, III, and IV are suppressed (Navarro and Boveris, 2007). The oxidants (i.e., H_2_O_2_) are produced (Petrosillo et al., 2008) while NADPH is insufficient (Pinto and Moraes, 2015; DeBalsi et al., 2017; Golpich et al., 2017). In addition, the high H_2_O_2_ levels alter the redox environment in cells and can be partially released into the cytoplasm through VDAC (Zou et al., 2018). Redox homeostasis is not only one of the characteristics of aging but also of neurodegenerative diseases, such as AD (Cenini et al., 2019). A clinical study on the quantitative detection of GSH in the occipital cortex showed that GSH levels in the elderly are significantly lower than those in the young (Emir et al., 2011). This age-dependent decline seems to be associated with cognitive impairment (Falls et al., 2018). Recent studies have shown that GSH depletion may cause metabolic stress in neurons by generating more ROS, which may eventually contribute to cognitive impairment (Gonzalez-Fraguela et al., 2018). As an important factor in regulating the energy-redox axis, NNT may also be a potential regulator in aging. Indeed, NNT overexpression can restore the mitochondrial NAD^+^ levels, enhance the reprogramming efficiency of senescent cells, and prolong the life-span of mesenchymal stem cells by delaying senescence (Son et al., 2016). Also, glucose metabolism is decreased and the life span is shortened in NNT^–/–^ mice (Kim et al., 2010). Therefore, there is a relationship between the TCA cycle and aging-related changes in the nervous system.

The majority of the ATP required by the brain is generated from the TCA cycle. Metabolomics analysis revealed that the utilization rate of glucose is altered in astrocytes extracted from 5xTg-AD mouse (van Gijsel-Bonnello et al., 2017). Several proteins and TCA cycle enzymes involved in glucose metabolism are also altered in AD brain tissue (Blass et al., 2000). Pyruvate is taken up into mitochondria via the mitochondrial pyruvate carrier, as the final metabolite of glycolysis (McCommis and Finck, 2015). Reduced mitochondrial pyruvate carrier activity might contribute to the inactivation of PDH caused by p-tau and alterative ketone body metabolism (Ding et al., 2013). Furthermore, pyruvate is the substrate for PDH and reduced in rodent models of AD (Sheu et al., 1985). Acetyl-CoA is made by PDH complex-catalyzed oxidative decarboxylation and flows into the TCA cycle. Acetyl-CoA levels in the synaptosomes of Tg2576 AD mice model are reduced (Bielarczyk et al., 2015). Moreover, acetyl-CoA is a substrate for the acetylation of the lysine group in AD pathological marker proteins, including β-site amyloid precursor protein (APP) cleaving enzyme 1 (BACE1) and APP (Jonas et al., 2010). Aβ causes a reduction in acetyl-CoA levels in neurons and glial cells (Bielarczyk et al., 2006). Both acetyl-CoA and succinyl-CoA are essential components for the formation of precursors of acetyl-choline, a neurotransmitter closely related to cognitive function (Kann and Kovacs, 2007). In addition, an examination of brains from deceased AD patients showed that the activities of the PDH complex, ICDH, and the α-KGDH complex are reduced (Sheu et al., 1994), while the activities of SDH and MDH are increased (Bubber et al., 2005). Citrate synthase (CS) activity appears to be negatively regulated by ApoE4 (Wilkins et al., 2017) and also decreased in AD patients (Fisar et al., 2016). As a downstream product of α-KG, succinyl-CoA may be reduced due to the downregulation of the pathway enzymes influencing the TCA cycle. However, there may be upregulation of succinate and aspartate (Zhou et al., 2018). Other enzymes and metabolites related to the metabolic process (e.g., citric acid, *cis*-aconitic acid, and fumaric acid) are decreased (Zheng et al., 2018). Thus, the changes in energy acquisition caused by these factors have various effects on many aspects of the body.

Under aerobic conditions, the TCA cycle is mainly active in mitochondria. Hence, the integrality of mitochondrial morphology and function are particularly essential for this process. Impaired mitochondria are observed in AD (Golpich et al., 2017) and lead to glucose hypometabolism, OXPHOS damage, excessive ROS accumulation, elevated oxidative stress (Wang et al., 2005), and disruption of the main pathway of glucose metabolism. Mitochondria are not only the main source of ROS but also the target of its attack. 8-oxoguanine (8-oxoG) accumulates in mtDNA (Bradley-Whitman et al., 2014) and the cytoplasm of hippocampal neurons (Song et al., 2011), which is an obvious sign of oxidative stress in AD patients. A vicious cycle occurs because oxidative stress can further aggravate mitochondrial dysfunction. The activity of the ETC complex is significantly reduced in AD (Holper et al., 2019), suggesting impaired OXPHOS. This phenomenon has been confirmed in mitochondria isolated from 3-month-old AD mice (Yao et al., 2009) and brain tissue from AD patients (Kim et al., 2000). The impairment of mitochondrial respiratory function in AD patients is also negatively correlated with Aβ levels and may be caused by the effect of Aβ on mitochondrial OXPHOS (Dragicevic et al., 2010). Similarly, another study demonstrated that Aβ could inhibit mitochondrial complexes I and IV (Bobba et al., 2013).

Mitochondria-driven glucose metabolism abnormalities have been recorded by magnetic resonance spectroscopy (MRS) or nuclear magnetic resonance (NMR) in several studies with AD rodent models. Decreased phosphomonoester levels and increased levels of phosphocreatine and adenosine diphosphate reflect changes in the oxidative metabolic rate, suggesting that oxidative stress occurs in the AD brain (Pettegrew et al., 1997). A reduction in glutamate, GSH, and GABA (Dedeoglu et al., 2004; Tiwari and Patel, 2012) suggests a damaged glutamatergic and GABAergic glucose oxidation and neurotransmitter cycle, which is also present in these mouse models. Thus, glutamine synthase impairment and decreased glutamate flux through the GABA pathway may be caused by mitochondrial dysfunction (Doert et al., 2015). The depletion of mitochondrial GSH leads to increased H_2_O_2_ levels and decreased mitochondrial membrane potential in neurons and astrocytes (Muyderman et al., 2007). Aβ can induce the internalization of the glutamate A2 (GluA2) subunit, which is highly Ca^2+^ impermeable and contributes to the production of proinflammatory cytokines by microglia that accelerates neurotoxicity in AD patients (Beppu et al., 2013; Noda, 2016). Therefore, altered levels of various enzymes and metabolites of the TCA cycle have an impact on mitochondrial function, resulting in abnormal redox homeostasis, reduced ATP production, and increased ROS production that injures surrounding tissues.

### Treatment

The cognitive impairment observed in AD can be improved by upregulating acetyl-CoA levels to attain normal mitochondrial function. This view is supported by experiments using CMS121 and J147 as candidate drugs against AD in mice (Currais et al., 2019) and long-term oral administration of acetyl-L-carnitine to AD patients, which provides additional acetyl-CoA (Epis et al., 2008). Specific therapeutic drugs for other metabolites require further research.

Nutraceuticals appear to be a feasible approach for protecting mitochondria. Micronutrients are key co-factors that sustain mitochondrial metabolic hemostasis, such as the generation of ATP, construction of the electron transport complex, and oxygen detoxification (Atamna, 2004). Thus, micronutrient deficiencies may cause drops in essential enzymatic activities and subsequently increase ROS production, decrease cellular energy metabolism, and aggravate mitochondrial abnormalities, promoting Aβ toxicity and AD progression (Young et al., 2007). Coenzyme Q10 (CoQ10), an antioxidant forming part of the ETC, can enhance mitochondrial function and promote ATP synthesis (Kumar and Singh, 2015). CoQ10 also inhibits nerve cell death resulting from oxidative stress and neurotoxins and has neuroprotective effects in double transgenic AD mouse models (Muthukumaran et al., 2018). Moreover, CoQ10 stabilizes mitochondria and reduces ROS production in fibroblasts from AD patients (Naderi et al., 2006; Ma et al., 2014; Muthukumaran et al., 2018). Thus, mitochondria-specific nutraceuticals (e.g., vitamins and CoQ10) can be beneficial for AD patients (Beal, 2004).

In summary, the TCA cycle and OXPHOS in the mitochondria can produce the greatest amount of energy for the body. The enzymes and metabolites involved in these reactions undergo various changes during aging and AD development. The imbalance in mitochondrial redox and resulting by-products (e.g., ROS) generated by these changes may cause inflammation in the surrounding tissues. Thus, to a certain extent, metabolic dysfunction could affect the occurrence and development of neurodegenerative disorders. However, most current treatments target symptom attenuation, and there is still no feasible research on specific treatments involving these altered intermediate products.

## Pentose Phosphate Pathway Impairment

### Normal Mechanism and Biological Function

The PPP is a significant component of intracellular oxidative catabolism, which is vitally important for oxidative stress resistance and the production of essential material for biological synthesis (Palmer, 1999; Russell et al., 1999). Although the pathway does not generate ATP, it can produce NADPH to maintain the reduced form of GSH (Cenini et al., 2019). When functioning as one of the most effective antioxidants, GSH is oxidized by ROS and is converted into the oxidized glutathione (GSSG), which subsequently enters a loop along with GSH peroxidase and GSH reductase.

The PPP is regulated by enzymes and the NADPH/NADP^+^ ratio. Alterations in either of the two factors will critically affect the pathway. A crucial enzyme system supports the phosphopentose pathway, among which G6P dehydrogenase (G6PD) and transketolase are particularly important. G6PD and transketolase are both rate-limiting enzymes, responsible for the redox equilibrium and the non-oxidative branch in the PPP, respectively (Gibson and Blass, 2007). In addition, transketolase plays a pivotal role in the material communication between glycolysis and the PPP (Kauffman, 1972). Moreover, an elevated NADPH/NADP^+^ ratio significantly inhibits G6PD, helping to disrupt G6P flux into the pathway. In conclusion, the PPP maintains redox hemostasis to prevent the initiation and development of oxidative stress in the brain.

### Altered PPP in Aging and AD

During aging, damaged mitochondria that produce less ATP and more ROS accumulate, leading to oxidative stress. G6PD plays a vital role in protecting neurons against endogenous ROS-mediated neurodegeneration in aging mice (Jeng et al., 2013). In the cerebral cortex of aged mice, there is a decline in the levels and activities of G6PD and other GSH-regenerating enzymes (Dukhande et al., 2009). Moreover, G6PD appears to be neuroprotective against endogenous ROS in the aged human brain (Sbodio et al., 2019). However, alterations in G6PD levels in the human brain have not been identified. Furthermore, decreased NADPase levels and increased NAD kinases (NADK) levels may alleviate oxidative stress during human aging by promoting the synthesis of NADPH and inhibiting the production of NADH (Clement et al., 2019).

Multiple risk factors, such as Aβ peptide, tau aggregation, and ApoE, play crucial roles in the PPP impairment contributing to AD. Oxidative stress and chronic inflammation are two critical factors demonstrated to trigger an elevation in the level of Aβ (Verdile et al., 2015) and subsequently the aggregation of toxic oligomers, particularly the Aβ_42_ forms. Later, aggregation of the Aβ peptides disturbs the redox balance, finally establishing a toxic cycle in AD patients (Hardy and Higgins, 1992; Musiek and Holtzman, 2015). Tau phosphorylation, aggregation, and accumulation are closely linked to APP malfunction, which will inhibit cellular metabolism, including the PPP (Takahashi et al., 2015; Kametani and Hasegawa, 2018). Mutant ApoE not only is involved in Aβ clearance and aggregation, but also reduces the rate of glucose metabolism before AD symptom onset (Reiman et al., 2001, 2005; Johnson et al., 2017), along with the low flux of glucose into the PPP.

NADPH, a reducing cofactor prominently recycled in the PPP, participates in the conversion of the oxidized form of GSH into the reduced form (Dringen, 2000). Hence, the PPP establishes a crucial relationship between glucose metabolism and the redox equilibrium. Notably, Aβ not only triggers ROS generation but also decreases GSH levels in astrocytes (Abramov et al., 2004; Canevari et al., 2004). These effects lead to disequilibrium and aggravate oxidative stress. Interestingly, various groups have found that there is increased glucose flux through the PPP to counteract Aβ toxicity (Hakim et al., 1976; Soucek et al., 2003; Sun et al., 2006; Allaman et al., 2010; de Bari et al., 2019). Research has identified a decreased level of ribose-5-phosphate and an elevated level of lactic acid, which indicates upregulation of the PPP (Oresic et al., 2011). Moreover, the dramatic degradation of phosphofructokinase B3 results in a higher level of glucose metabolism via the PPP than via glycolysis in neuronal cells (Herrero-Mendez et al., 2009).

Accumulating evidence indicates that there is an alteration of the enzymes in the phosphate pentose shunt, which could significantly impact the antioxidant system in AD. G6PD is involved in generating NADPH for the reduction of the oxidized GSH, which helps to maintain the redox hemostasis. Several studies reported elevated levels of sulfhydryls and upregulation of G6PD in AD (Martins et al., 1986; Russell et al., 1999; Scheltens et al., 2016), which may be neuroprotective. However, the mechanism underlying this protective effect remains elusive (Tang, 2019). Notably, another report found that G6PD activity was decreased in the hippocampi of human AD brains (Bigl et al., 1999). In addition to the enzymatic activity, antioxidants are generated in astrocytes to resist oxidative stress (Ben-Yoseph et al., 1994; Rahman et al., 2000). In response to the aggregation of Aβ, ROS levels increase and there is a decrease in GSH in astrocytes in both AD and mild cognitive impairment (Sultana et al., 2008; Mandal et al., 2012).

Furthermore, the activities of transketolase and their common coenzymes thiamine diphosphate, GSH peroxidase, glutathione-S-transferase, and δ-aminolevulinate dehydratase (δ-ALA-D) have all been demonstrated to be decreased in AD (Sheu et al., 1988; Mastrogiacomo et al., 1996; Ansari and Scheff, 2010; Yu et al., 2018). Multiple metabolites altered by ApoE are identified within the PPP, including gluconolactone, gluconate, and G6P (Johnson et al., 2017). These findings may provide a novel strategy to increase GSH production by regulating the activity of these enzymes in the PPP.

### Treatment

AD therapies concentrate on oxidative stress, mainly targeting the PPP. For example, Krautwald and Munch (2010) emphasized that advanced glycation end products and their methylglyoxal precursors are both biomarkers and pathogenic factors in AD, with direct neurotoxicity related to oxidative stress and apoptosis. Meanwhile, they proposed that the formation of advanced glycation end products occurred through a lower methylglyoxal concentration, which could be achieved by permitting a higher flux through the PPP. Cai et al. (2020) hypothesized that hollow manganese Prussian white nanocapsules (HMPWCs) participated in the resistance to the harmful effects of tau by relieving neuronal inflammation, eliminating ROS, and inhibiting tau hyperphosphorylation. Targeting of astrocytic NRF2, a regulator of GSH synthesis, could be a potent therapeutic strategy in AD (Oksanen et al., 2019).

Because NADPH is essential for reducing GSSG to GSH, PPP is a pivotal part of the oxidative stress observed in AD pathology. Nevertheless, the upregulation of glucose flux into the PPP increases the synthesis of NADPH. However, it is controversial whether the activity of G6PD is activated or suppressed in AD. Hence, it is uncertain whether NADPH production is increased or decreased, and further investigation is needed. Despite the uncertain mechanism, there are still strategies to relieve PPP dysfunction and oxidative stress in AD.

## Conclusion and Future Perspectives

**FIGURE 1:**
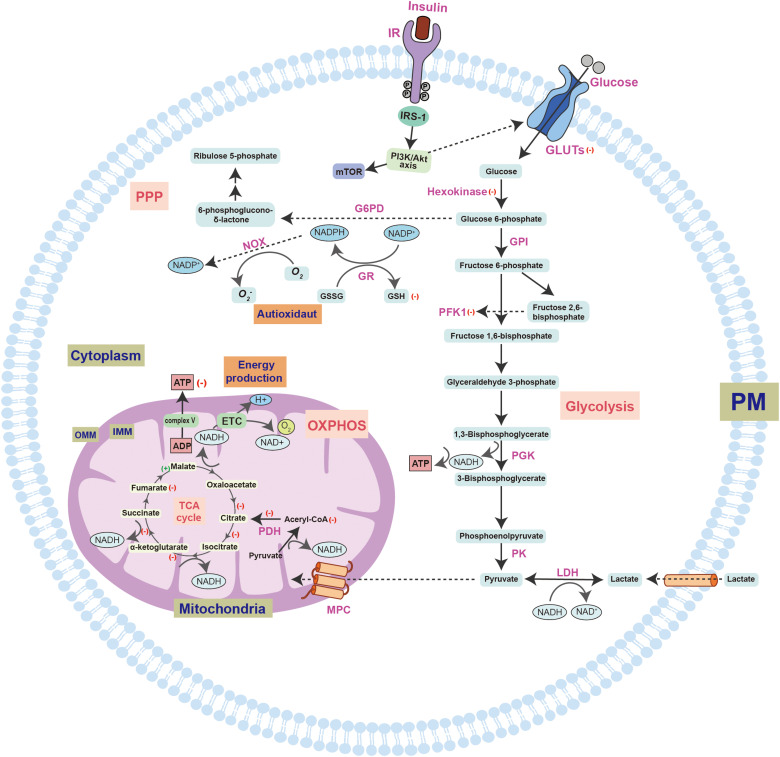
A schematic overview of insulin signaling and glucose utilization pathways in the brain. Glucose enters the cell through the synergistic action of a variety of GLUTs and is further catalyzed by different enzymes. However, the most important factor regulating glucose transport is insulin and its receptors. Insulin promotes the activation of IR through autophosphorylation of specific tyrosine residues, which can bind to IRS-1/2 and activate it, thereby regulating the cascade of energy metabolism signals, such as the PI3K-Akt-mTOR signal pathway. In the brains of early AD patients with MCI, alterations in the gene expression profiles will lead to increased insulin consumption. The symptoms of cerebral diabetes, such as decreased glucose metabolism and energy exhaustion, have been found in MCI samples. The decrease in glucose metabolism may aggravate the impairment of cognitive function. Furthermore, the brain in the early stage of AD under diabetic conditions can aggravate the symptoms of peripheral diabetes. The biochemical diagram of the changes in normal intracellular glucose catabolism and ATP synthesis includes the glycolysis pathway that initially occurs in the cytoplasm, the TCA cycle and OXPHOS pathway that occurs in the mitochondria, and the PPP, which provides raw materials for biosynthesis and regulates redox. Changes in these biological processes in the AD brain are shown by the following symbols: (+), increased expression; (–), decreased expression. The structural and functional disorders of mitochondria eventually lead to decreased ATP production, increased ROS production, and the occurrence of oxidative stress. Moreover, further mitochondrial damage will also lead to a vicious cycle that aggravates neurodegeneration. GLUT, glucose transporter; G6PD, glucose 6-phosphate dehydrogenase; F6BP, Fructose 6-bisphosphate; PFK1, phosphofructokinase 1; PGK, phosphoglycerate kinase; LDH, lactate dehydrogenase; PK, pyruvate kinase; MPC, mitochondrial pyruvate carrier; ETC, electron transport chain; PDH, pyruvate dehydrogenase; OXPHOS, oxidative phosphorylation; TCA, tricarboxylic acid; IMM, inner mitochondrial membrane; OMM, outer mitochondrial membrane; NADP+, nicotinamide adenine dinucleotide phosphate; GR, glutathione reductase; NOX, NADPH-oxidase; GSH, glutathione; GSSG, glutathione; O2-, superoxide anion. (+): indicates an increase in expression, (–): indicates a decrease in expression.

In this review, we summarized the metabolic deficits, including glucose metabolism dysregulation, glycolysis dysfunction, and PPP impairment, in AD (Figure 1). These deficits cause significant effects on AD pathogenesis. One direct consequence of these deficits is the inhibition of ATP generation, which leads to insufficient energy to support the normal neuronal functions and, ultimately, neurodegeneration. In addition, the metabolic deficits indirectly trigger neuronal death via mitochondrial dysfunction, oxidative stress (increased ROS, decreased NADPH), and inflammation.

Compared to previous review articles (Atamna and Frey II, 2007; Yin et al., 2016; Butterfield and Halliwell, 2019), we not only described changes in the regulation of signal pathways but also discussed in detail, the changes of implicated enzymes and metabolites during energy metabolism in AD. Moreover, we highlighted the intimate relationship between AD pathogenesis and glucose metabolism dysregulation, implying that a focus on the preclinical and clinical manifestations of glucose metabolic dysregulation in AD might be a promising strategy to diagnose and prevent or slow the progression of this disease.

Peripheral diabetes appears to aggravate these metabolic changes rather than cause them. Moreover, peripheral diabetes-related abnormalities do not directly influence the expression of diabetes-related genes in the brain (Hokama et al., 2014), suggesting that AD pathology may play a key role in altering gene expression, which is correlative with diabetes in AD patients. The GLUT isoforms have variable effects on glucose utilization. Although altered cerebral glucose uptake is currently considered a predictive method for diagnosing AD, there is still a lack of adequate research to support its widespread use. Because of the constantly changing pathological processes in AD (Varma et al., 2018), the metabolite signal changes in the blood cannot reflect the changes in the brain in time, and there is still no accurate and easy-to-use index for preclinical and clinical diagnosis and treatment. Thus, it is difficult to assess whether peripheral signals related to the state of the disease are also reflected in the brain. The combination of multi-omics analysis and multi-type data will increase our understanding of the profound mechanisms of AD and identify potential biomarkers for diagnosis, prognosis and monitoring treatment of this disease. Therefore, further research is needed to develop more accurate and convenient diagnostic techniques for clinical use.

Alzheimer’s disease treatment currently concentrates on symptom attenuation, targeting the specific enzymes or intermediate products. Different types of medications are used to recover the normal function of the proteins or ameliorate the intermediate outcomes, including some exogenous antioxidant interventions (vitamin E, polyphenol, and deuterated lipids). Although symptom treatment is the most widespread method for managing AD, it is not the best strategy to slow or reverse the neuronal degeneration because it only targets the manifestations of the disease and not its essence. Targeting these pathways may lead to the development of effective treatments (Table 1). We suggest that future AD treatments focus on early metabolic changes, clinical predictions, diagnosis, prevention, and the combined treatment of multiple pathogens at the pre-clinical and clinical stages using personalized drugs to prevent or delay the progression of AD. Further efforts are still needed to understand the metabolic basis of the etiology and pathogenesis of AD.

**TABLE 1:** Defective metabolic pathways and treatments in AD.

Defective metabolic pathways	Potential treatments	Outcomes	References
A decreased level of GLUT1 and GLUT3			Simpson et al., 1994; An et al., 2018
The downregulation of hexokinase triggered by the increased G6P competitively binding with hexokinase	Decrease the concentration of G6P	Increase the activity of hexokinase in order to facilitate glycolysis	Liu et al., 1999; Demarest et al., 2020
The inhibition of neuroprotective effects of 2DG caused by an increased concentration of NADH	Control the activity of GAPDH	Decrease the concentration of NADH and promote 2DG to inhibit the effect of Aβ on neuronal cells	Vilalta and Brown, 2014; Kim et al., 2017; Shen et al., 2017
A decreased level of acetyl-CoA	Treat with acetyl-L-carnitine which provides additional acetyl-CoA	Upregulate the activity of acetyl-CoA	Epis et al., 2008; Bielarczyk et al., 2015
A decreased level of PDH, ICDH, α-KGDH			Sheu et al., 1985; Sheu et al., 1994
An increased level of SDH (complex II) and MDH			Bubber et al., 2005
A decreased level of CS			Fisar et al., 2016
A decreased level of citric acid, cisAconitic acid, fumaric acid			Zheng et al., 2018
A lower flux of glucose into the PPP resulted from tau aggregation and ApoE mutation	Treat with Hollow manganese Prussian white nanocapsules	Relieve neuronal inflammation, eliminate ROS, and inhibit tau hyperphosphorylation.	Cai et al., 2020
An increased or decreased level of G6PD and NADPH			Martins et al., 1986; Russell et al., 1999; Scheltens et al., 2016; Tang, 2019

## Author Contributions

XY and XZ contributed to the conceptualization and methodology. XY, YH, BW, SW, and XZ wrote the first draft of the manuscript. YH created the figure. BW prepared the table. All the authors approved the final manuscript.

## Conflict of Interest

The authors declare that the research was conducted in the absence of any commercial or financial relationships that could be construed as a potential conflict of interest.
